# Cardiovascular risk associated with chronic treatment of paliperidone, olanzapine, risperidone and aripiprazole

**DOI:** 10.1192/j.eurpsy.2024.449

**Published:** 2024-08-27

**Authors:** A. L. Montejo, C. Bermejo, J. Matías, T. Martín, J. Matías-Polo, Y. Santana, J. López-López, R. de Alarcón, J. M. Acosta

**Affiliations:** ^1^Psychiatry, University of Salamanca. IBSAL; ^2^Neurociencias, IBSAL; ^3^Psychiatry, Complejo Asistencial Salamanca. IBSAL, Salamanca, Spain

## Abstract

**Introduction:**

Weight gain, QT interval prolongation, and dyslipidemias associated with the chronic use of some antipsychotic medications can explain a higher prevalence of cardiovascular risk in these psychiatric population. The D’Agostino Index include some factors such as age, total cholesterol, high-density lipoproteins, systolic blood pressure increased, antihypertensive treatment, smoking, and diabetes, to estimate an individual’s risk (low, moderate or severe) of developing a cardiovascular event through a period of 10 years or throughout the patient’s lifetime.

**Objectives:**

To compare the degree of cardiovascular risk using the D’Agostino Index, among different antipsychotic medications.

**Methods:**

An estimation of cardiovascular risk (low, moderate, or high) was performed with the D´Agostino index in a sample of 144 patients (82 men and 62 women) mean age 45,2 +/- 10.13. All patients were treated for at least one year at a therapeutic dose and adhered to their treatment regimen correctly. Subjects with some relevant pre-existing unstable heart disease were excluded. All patients previously provided informed consent and were of legal age. Clinical data on medical history, concomitant medications, and risk factors were collected. A completed physical exam, waist circumference, lab sample, a lifestyle scale, and an evaluation of vital signs in accordance with European Society of Hypertension were evaluated. Statistical analysis was carried out using the statistical software SPSS version 26.0. A significance level α=0.05 was considered throughout the study.

**Results:**

The four most consumed antipsychotics were risperidone 9.72% (n=14), paliperidone 25.7% (n=37), olanzapine 14.6% (n=21), and aripiprazole 34.7% (n=50). Descriptively, it was observed that the drugs most associated with moderate or high risks were paliperidone (37.8%) and olanzapine (33.3%), risperidone (28.6 %). Aripiprazol (22%) was the less associated compound with moderate/high cardiovascular risk.

**Conclusions:**

Subjects treated with olanzapine and paliperidone showed a higher association with cardiovascular risk. Predicting cardiovascular risk could provide individual benefits by enabling lifestyle modifications, pharmacological treatment changes, or closer monitoring to reduce cardiovascular risk.

**Disclosure of Interest:**

A. Montejo Grant / Research support from: This study has been funded by the Instituto de Salud Carlos III (ISCIII) through the project PI19/1596 and co-funded by the European Union., C. Bermejo: None Declared, J. Matías: None Declared, T. Martín: None Declared, J. Matías-Polo: None Declared, Y. Santana: None Declared, J. López-López: None Declared, R. de Alarcón: None Declared, J. Acosta: None Declared

